# Signs and symptoms of carriers of non-*DMD* X-linked neuromuscular diseases: A scoping review

**DOI:** 10.1177/22143602251330441

**Published:** 2025-03-29

**Authors:** Job Simons, Amanda Dekker, Rosanne Govaarts, Anna Sarkozy, Christian Windpassinger, Saskia Houwen, Nicol Voermans

**Affiliations:** 1 Department of Neurology, Donders Institute for Brain, Cognition and Behaviour, Radboud University Medical Center, Nijmegen, The Netherlands; 2 Department of Rehabilitation, Amalia Children's Hospital, Radboud University Medical Center, Nijmegen, The Netherlands; 3 Department of Radiology, C.J. Gorter MRI Center, Leiden University Medical Center, Leiden, The Netherlands; 4 Dubowitz Neuromuscular Centre, UCL Great Ormond Street Institute of Child Health, London, UK; 5 Diagnostic and Research Institute of Human Genetics, Medical University of Graz, Graz, Austria; 6 Neurogenetics Laboratory, Department of Neurology, Medical University of Graz, Graz, Austria

**Keywords:** neuromuscular diseases, signs and symptoms, genetic diseases, X-linked, myopathies, structural, congenital, heterozygote

## Abstract

**Background:**

It has been known for long that females carrying pathogenic variants in the *DMD* gene often report symptoms and/or exhibit signs of the disease. However, a notable knowledge gap exists concerning the signs and symptoms of female carriers of other X-linked neuromuscular diseases (XLNMDs).

**Objective:**

This scoping review aims to provide a comprehensive outline of existing literature regarding the signs and symptoms of carriers of non-*DMD* XLNMDs to raise awareness among both researchers and clinicians.

**Methods:**

Three electronic databases were used for the literature search (PubMed, Embase, Web of Science). Studies on the signs and symptoms of carriers of non-*DMD* XLNMDs were included.

**Results:**

We included 44 articles for this review with a total of 354 carriers of non-*DMD* XLNMDs (mean age 43.9 years, std. deviation 17.4). Muscular signs and symptoms were reported for 125 carriers (X-linked myotubular myopathy (XLMTM): n = 96 (65%); Kennedy's disease (KD): n = 25 (32%); X-linked recessive Charcot-Marie-Tooth disease (CMTXR): n = 2 (15%); Uruguay faciocardiomusculoskeletal syndrome (FCMSU): n = 1 (33%); Barth syndrome (BS): n = 1 (100%)). In terms of ancillary investigations, abnormalities in histopathology and imaging were the most frequent with 44 carriers having abnormalities found by these testing (XLMTM: n = 36 (24%); Emery-Dreifuss muscular dystrophy 1 (EDMD1): n = 4 (5%); KD: n = 4 (5%) / XLMTM: n = 18 (12%); EDMD1: n = 1 (1%); KD: n = 5 (6%); X-linked myopathy with postural muscle atrophy (XMPMA): n = 19 (83%); BS: n = 1 (100%)). A difference between the number of EDMD1 carriers with cardiovascular signs and symptoms (n = 2 (1%)) and the number of carriers with abnormal electrocardiography tests (n = 20 (23%)) was also noted.

**Conclusion:**

Carriers of non-*DMD* XLNMDs exhibit a variety of signs and symptoms that could impact quality of life, making it vital for clinicians to be aware of these patients.

## Introduction

A number of neuromuscular diseases (NMDs) have an X-linked (XL) recessive inheritance pattern, causing female heterozygous carriers to traditionally be considered asymptomatic. However, it has gradually become evident that carriers of XLNMDs can exhibit symptoms. Duchenne muscular dystrophy (DMD) and Becker muscular dystrophy (BMD) are well-known XLNMDs with severely affected male patients. Carriers of a pathogenic variant in the *DMD* gene experience a wide range of symptoms and are now preferably referred to as female patients with DMD or BMD.^1,2^

The presence of symptoms among female patients with DMD or BMD raised our interest in symptoms of carriers of other XLNMDs. X-linked myotubular myopathy (XLMTM) for example, is a severe neuromuscular disease with a prevalence of about 1 in 50,000 new-born males. The average life expectancy of these patients is less than 30 months.^3^ XLMTM carriers may also experience symptoms, ranging from minimally manifesting to being severely affected.^4^ Symptoms described include limb girdle, axial and respiratory weakness.^5^ This finding highlighted the importance of recognizing symptomatic carriers and how their symptoms could influence their day-to-day life significantly.

The objective of this scoping review is to explore the signs and symptoms of carriers of non-*DMD* XLNMDs and identify possible knowledge gaps. The ultimate goal is to estimate the impact these signs and symptoms have on the lives of carriers and thus increase awareness among both researchers and clinicians.

## Materials and methods

The scoping review was conducted in accordance with the Preferred Reporting Items for Systematic Reviews and Meta-Analysis extension for Scoping Reviews (PRISMA-ScR) guidelines. These guidelines offer a structured framework for systematically mapping diverse and often complex evidence in order to identify the knowledge gaps in a certain field.^6^ The aim of this methodology is to create a comprehensive overview of the available evidence related to carriers of non-*DMD* XLNMDs. A study protocol for this scoping review was registered at Open Science Framework (OSF), which can be found via the registration DOI: https://doi.org/10.17605/OSF.IO/EGKFH.

### Sources and search strategy

A literature search was conducted including the following databases: PubMed (MEDLINE database), Embase (Ovid) and Web of Science (Clarivate Analytics). This search was done on the 22nd of September, 2023, without limitations on the year of publication. The search strategy incorporated MeSH, Emtree and title and abstract terms. The included non-*DMD* XLNMDs were derived from expert opinion, the RadboudUMC exome panel and various sources on the internet.^7,8^ The search strategy was formulated with the help of an information specialist at the Medical Library of RadboudUMC in Nijmegen. In Supplement 1, the full search strategy with all its included XLNMDs can be found.

### Selection criteria

Any article with information on the signs and/or symptoms of carriers of non-*DMD* XLNMDs was included, without restriction on the year of publication. Only biological females were included and XL-dominant diseases were excluded. For example, certain FHL1 mutations that were described as or associated with a dominant inheritance were excluded. Articles without full text available or articles that were not in Dutch or English were excluded and literature reviews were used for forward and backward citation in order to prevent duplications in the dataset.

All results that derived from the search strategy in each individual database were collected in EndNote20 and duplicates were removed. Records were then screened on title and abstract by three independent reviewers (JS, AD, SH) using software from Rayyan Systems Inc.^9^ After the screening process, full text of the selected articles was read and examined for eligibility.

### Critical appraisal

The selected articles were assessed on quality using a critical appraisal format as shown in Supplement 2. This format was developed by modifying the Joanna Briggs Institute (JBI) critical appraisal tools for ‘Case Reports,’ ‘Case Series,’ and ‘Analytical Cross-Sectional Studies’ (https://jbi.global/critical-appraisal-tools). The articles were evaluated based on nine criteria: a clear description of the inclusion criteria, appropriate and well-defined recruitment of study participants, the use of standardized and reliable measurement tools, the application of valid measurement methods, the reporting of patient demographics (including at least age), the provision of relevant clinical information, the use of appropriate statistical analyses, the presence of statistically significant results and, finally, whether the conclusions were justified by the findings. Articles were assessed using the categories ‘yes’, ‘no’, ‘unclear’, or ‘not applicable’. The final score was determined by dividing the total number of ‘yes’ responses by the number of criteria applicable to the study design of the specific article, creating a score with 0 as the lowest and 1 as the highest value. Quality assessment was performed independently by two reviewers (JS and AD). Any disagreements regarding the quality assessment were deliberated among the reviewers until agreement was reached.

### Data charting

A data-extraction sheet was made containing article ID, key information, patient number, age of each patient, diagnosis and the signs and/or symptoms they exhibit. *Online Mendelian Inheritance in Man (OMIM)* codes were noted for each article. If an article did not specify the gene mutation of the XLNMD, the mutation with the highest prevalence was presumed. Data was then extracted by two independent reviewers (JS and AD). Signs and symptoms were defined as traits of physical examination and patient complaints. Findings of ancillary investigations were defined as objectively measured clinical tests. Signs and symptoms were divided into muscular, skeletal and ligamental, respiratory, cardiovascular, developmental delay and functional impairment and lastly, other signs and symptoms. Findings of ancillary investigations were divided into histology, imaging, respiratory tests, electrocardiography, electrophysiology and laboratory investigations. The full data extraction sheet can be found in Supplement 3.

### Statistical analysis

Descriptive statistics were used to provide an overview of the signs, symptoms and findings of ancillary investigation of carriers of non-*DMD* XLNMDs. The proportion of carriers with abnormal findings in relation to the total number of carriers of each XLNMD was displayed in a bar diagram using percentages.

## Results

**Figure 1.**
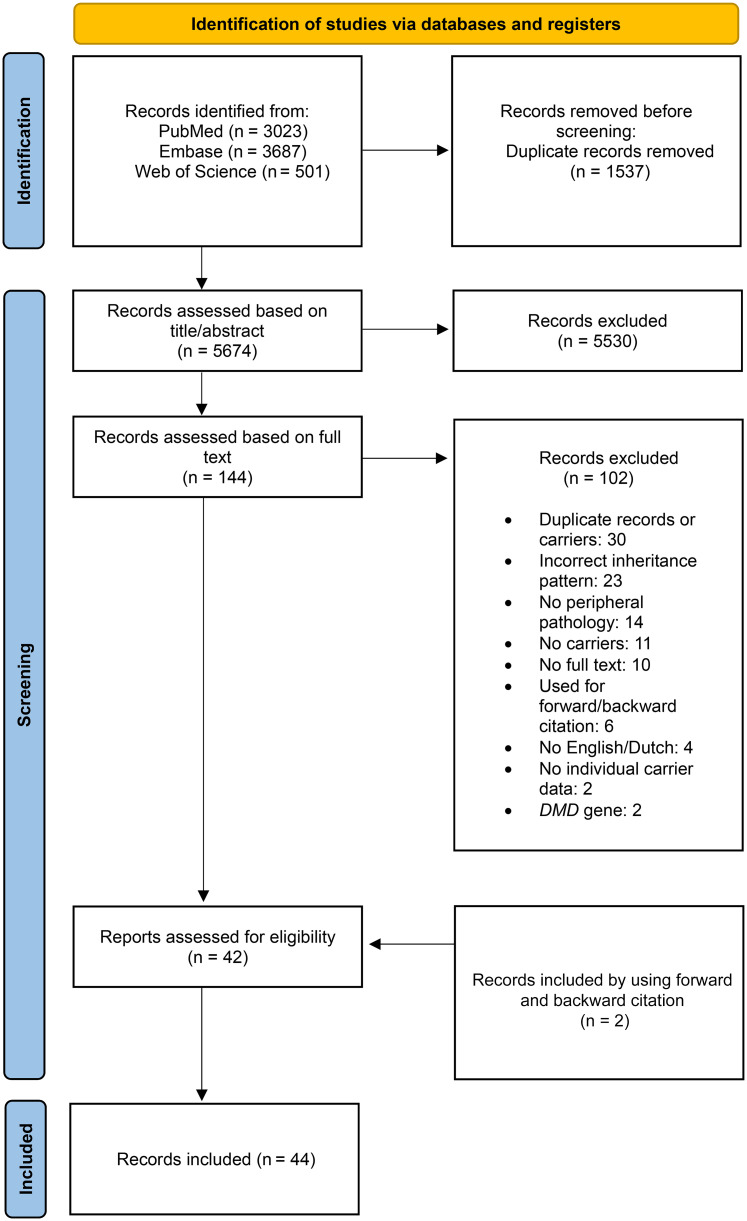
PRISMA flow diagram visualizing the selection procedure of records for this scoping review.

The search strategy identified 7211 articles (PubMed: n = 3023; Embase: n = 3687; Web of Science: n = 501). After the removal of duplicate publications and the title and abstract screening, 144 articles remained. Articles were then assessed on full text and eligibility, which resulted in 44 studies, including the two articles that were included after forward and backward citation. The selection process can be found in the PRISMA flow diagram in Figure 1.^10^

The 44 articles resulted in data on 354 carriers. 147 women were carrier of XLMTM (*OMIM 310400*), 88 of Emery-Dreifuss muscular dystrophy 1 (EDMD1, *OMIM 310300*), 78 of Kennedy's disease (KD, *OMIM 313200*), 23 of X-linked myopathy with postural muscle atrophy (XMPMA, *OMIM 300696*), 13 of X-linked recessive Charcot-Marie-Tooth disease (CMTXR, *OMIM 302801/2*) and three of Uruguay faciocardiomusculoskeletal syndrome (FCMSU, *OMIM 300280*). Lastly, there was one carrier of Barth syndrome (BS, *OMIM 302060*) and one of X-linked myopathy with excessive autophagy (XLMEA, *OMIM 310440*). The mean age of all carriers was 43.9 years old (std. deviation 17.4). Critical characteristics of the included articles can be found in Table 1. The results of the critical appraisal can be found in Supplement 2. All assessed articles were considered to be of sufficient quality by both reviewers. Therefore, no articles were excluded based on the critical appraisal.

**Table 1.** Overview of included articles.

Article ID	First author Year Country	XLNMD (*OMIM* gene abbreviation)	Sample size (share of symptomatic carriers in %)	Mean age (years)	Study objective	Reported signs/symptoms and findings of ancillary investigations (full list in Supplement 3)
1	Bevilacqua 2008 FR	XLMTM (MTM1)	3 (100%)	35	To investigate the histological features in patients and carriers of XLMTM	Muscle weakness, delayed motor milestones, muscle fiber abnormalities, imaging abnormalities, restricted vital capacity
2	Bialer 1991 US	EDMD1 (EDMD1)	34 (18%)	Unknown	To investigate cardiac symptoms in patients and carriers of XLEDMD	ECG abnormalities
3	Boriani 2003 IT	EDMD1 (EDMD1)	3 (100%)	57	To characterize cardiac manifestations in patients and carriers of XLEDMD	ECG abnormalities, lightheadedness
4	Koutsis 2015 GR	KD (SMAX1)	8 (25%)	Unknown	To investigate the clinical traits of a KD cohort, including carriers	Muscle cramps
5	Gómez-González 2021 ES	XLMTM (MTM1)	1 (100%)	13	To describe the genotype of a patient with suspected XLMTM	Muscle weakness, fatigue, reduced mobility, decreased autonomy, lumbar scoliosis, muscle fiber abnormalities
6	Canki-Klain 2000 HR	EDMD1 (EDMD1)	1 (100%)	45	To investigate the clinical symptoms of a family affected by XLEDMD, including carriers	Palpitations, ECG abnormalities
7	Carboni 2012 IT	EDMD1 (EDMD1)	7 (14%)	40	To assess the cardiac and muscle involvement of a family affected by XLEDMD, including carriers	ECG abnormalities
8	Chen 1999 TW	KD (SMAX1)	5 (40%)	24	To clinically and genetically analyze a family affected by KD, including carriers	Muscle cramps, tremor, fasciculations, decreased reflexes,
9	Ishihara 2001 JP	KD (SMAX1)	8 (88%)	58	To investigate the relation between phenotype and X-inactivation in KD carriers	Muscle cramps and weakness, EMG abnormalities, CK elevation
10	Mariotti 2000 IT	KD (SMAX1)	19 (79%)	43	To characterize clinical and histological manifestations in patients and carriers of KD	Muscle cramps, fasciculations, altered neurophysiology
11	Oldfors 1989 SE	XLMTM (MTM1)	2 (100%)	40	To investigate clinical and histological symptoms in a family affected by XLMTM, including female carriers	Muscle fiber abnormalities
12	Schara 2003 GE	XLMTM (MTM1)	1 (100%)	5	To report the case of a female patient affected by XLMTM	Muscle weakness, hypotonia, dyspnea, abnormal reflexes, muscle fiber abnormalities
13	Viggiano 2019 IT	EDMD1 (EDMD1)	30 (17%)	40	To investigate the relation between cardiac phenotype and X-inactivation in XLEDMD carriers	ECG abnormalities
14	Jääskeläinen 2002 FI	XLMEA (XMEA)	1 (100%)	55	To investigate clinical and electrophysiological features and muscle MRI in patients and one carrier of XLMEA	Difficulty walking on the heels
15	Fishbein 1993 US	EDMD1 (Presumably EDMD1)	1 (100%)	45	To report the case of a carrier of XLEDMD who experienced sudden death	Sudden death, ECG abnormalities, imaging abnormalities
16	Fryns 1980 BE	CMTXR (Unknown, CMTX1-6)	4 (100%)	54	To characterize clinical and electrophysiological symptoms of CMTXR carriers	Pes cavus, altered neurophysiology
17	Cocanougher 2019 US	XLMTM (MTM1)	11 (82%)	45	To investigate the clinical symptoms, pulmonary and cardiac function, and muscle imaging in XLMTM carriers	Muscle weakness, imaging abnormalities, decreased FVC
18	García-García 2018 ES	XLMTM (MTM1)	1 (100%)	55	To report the case of a female patient affected by XLMTM	Muscle weakness, exercise intolerance, nocturnal dyspnea, decreased FVC, altered neurophysiology, imaging abnormalities
19	Felice 2018 US	XLMTM (MTM1)	1 (100%)	58	To report the case of a female patient affected by XLMTM	Muscle weakness, muscle atrophy, contractures, CK elevation, decreased FVC, altered neurophysiology, muscle fiber abnormalities
20	Zhao 2018 CN	XLMTM (MTM1)	2 (100%)	25	To characterize symptoms, imaging, muscle biopsies and genetic analysis in a cohort of patients and carriers with various CNMs	Muscle weakness, contractures, abnormal reflexes, muscle fiber abnormalities, imaging abnormalities
21	Tanner 1999 CH	XLMTM (MTM1)	3 (33%)	53	To describe the clinical characteristics and X-inactivation pattern of a female patient affected by XLMTM	Muscle weakness, kyphoscoliosis, pes equinovarus, altered neurophysiology, muscle fiber abnormalities
22	Sutton 2001 GB	XLMTM (MTM1)	1 (100%)	29	To report the case of a female patient affected by XLMTM	Muscle weakness, scoliosis, urinary incontinence, CK elevation, altered neurophysiology, muscle fiber abnormalities
23	Sorarù 2008 IT	KD (SMAX1)	3 (100%)	41	To characterize symptoms and muscle biopsies in patients and carriers of KD	Muscle hypertrophy, altered neurophysiology, muscle fiber abnormalities
24	Nevo 1999 US	EDMD1 (EDMD1)	1 (100%)	Unknown	To detect defects in the STA gene in three XLEDMD families	Abnormal cDNA
25	Grogan 2005 US	XLMTM (MTM1)	11 (36%)	45	To investigate symptoms in carriers of XLMTM within two families	Muscle weakness, skeletal asymmetry, imaging abnormalities, muscle fiber abnormalities
26	Biancalana 2017 FR	XLMTM (MTM1)	17 (100%)	37	To investigate clinical symptoms, histology, imaging and molecular data in XLMTM carriers	Muscle weakness, contractures, respiratory symptoms, difficulties running, scoliosis, muscle fiber abnormalities, decreased VC, CK elevation
27	Cosson 2012 FR	BS (BTHS)	1 (100%)	3	To report the case of a female patient affected by BS	Muscle weakness, heart failure, respiratory symptoms, imaging abnormalities, ECG abnormalities, neutropenia
28	Dahl 1995 FR	XLMTM (MTM1)	1 (100%)	5	To report the case of a female patient affected by XLMTM	Hypotonia, delayed psychomotor development, muscle fiber abnormalities
29	Emery 1987 US	EDMD1 (Presumably EDMD1)	7 (57%)	45	To review the signs and symptoms in multiple families with XLEDMD	CK elevation, ECG abnormalities
30	Souza 2020 BR	XLMTM (MTM1)	6 (67%)	54	To examine phenotypic variation in manifesting carriers of XLMTM and identify potential genetic modifiers	Muscle weakness, cramps and atrophy, walking difficulties, respiratory symptoms
31	Savarese 2016 IT	XLMTM (MTM1)	4 (100%)	36	To perform genetic analyses and investigate signs and symptoms in patients and carriers of XLMTM	Muscle weakness, difficulties climbing stairs, reduced reflexes, scoliosis, CK elevation, imaging abnormalities, altered neurophysiology
32	Meriggioli 1999 US	KD (SMAX1)	6 (17%)	49	To describe clinical and electrophysiological features in patients and one carrier of KD	Muscle weakness, cramps and atrophy, fasciculations, reduced reflexes, altered neurophysiology
33	Hammans 2000 GB	XLMTM (MTM1)	4 (25%)	57	To report the case of a female patient affected by XLMTM	Muscle weakness, urinary incontinence, dysphagia, altered neurophysiology, muscle fiber abnormalities
34	Quadrelli 2000 UY	FCMSU (FCMSU)	3 (33%)	Unknown	To report signs and symptoms of patients and carriers of FCMSU within one family	Prominent muscular development, scoliosis, CK elevation
35	Helliwell 1998 GB	XLMTM (MTM1)	1 (100%)	0	To investigate clinical and histological features in patients and one carrier of XLMTM	Hypotonia, poor respiratory effort, muscle fiber abnormalities
36	Jungbluth 2003 GB	XLMTM (MTM1)	1 (100%)	6	To report the case of a female patient affected by XLMTM	Muscle weakness, delayed motor milestones, fecal and urinary incontinence, decreased FVC, muscle fiber abnormalities, imaging abnormalities
37	Mora 1997 IT	EDMD1 (EDMD1)	1 (100%)	Unknown	To investigate the use of emerin expression as a diagnostic tool for XLEDMD	Abnormal immunostaining
38	Guber 2017 US	KD (SMAX1)	13 (38%)	Unknown	To determine prevalence and features of fatty liver disease in patients and carriers of KD	Imaging abnormalities
39	Manilal 1997 GB	EDMD1 (EDMD1)	3 (100%)	Unknown	To demonstrate analysis of leucocytes and skin as a diagnostic tool for XLEDMD	Abnormal immunohistochemistry
40	Sobue 1993 JP	KD (SMAX1)	8 (50%)	37	To investigate the subclinical phenotype in female carriers of KD	Altered neurophysiology, muscle fiber abnormalities
41	Reumers 2021 NL	XLMTM (MTM1)	76 (64%)	47	To characterize the clinical features in patients and carriers of XLMTM	Muscle weakness, fatigue, muscle cramps, respiratory weakness
42	Binder 2012 AT	XMPMA (XMPMA)	23 (100%)	52	To analyze the cardiac phenotype of patients and carriers of XMPMA	Contractures, NT-proBNP elevation, ECG abnormalities, imaging abnormalities
201^a^	Pareyson 1995 IT	KD (SMAX1)	8 (0%)	Unknown	To investigate clinical and molecular signs and symptoms in two families with KD, including carriers	None
202^a^	Ionasescu 1992 US	CMTXR (Presumably CMTX2 and CMTX 3)	9 (0%)	Unknown	To clinically and genetically study two families with CMTXR, including carriers	None

^a^ Article IDs of articles with asymptomatic carriers (see also Supplement 3); Abbreviations: XLNMD = X-linked neuromuscular disease; OMIM = Online Mendelian Inheritance in Man; XLMTM = X-linked myotubular myopathy; EDMD1 = Emery Dreifuss muscular dystrophy 1; KD = Kennedy's disease; XLMEA = X-linked myopathy with excessive autophagy; CMTXR = X-linked recessive Charcot-Marie-Tooth disease; BS = Barth syndrome; FCMSU = Uruguay faciocardiomusculoskeletal syndrome; XMPMA = X-linked myopathy with postural muscle atrophy; CNM = centronuclear myopathy; MRI = magnetic resonance imaging; STA = emerin gene; ECG = electrocardiogram; EMG = electromyography; CK = creatine kinase; FVC = forced vital capacity; VC = vital capacity; cDNA = copy DNA; NT-proBNP = N-terminal pro-B-type natriuretic peptide.

### Signs and symptoms

**Figure 2.**
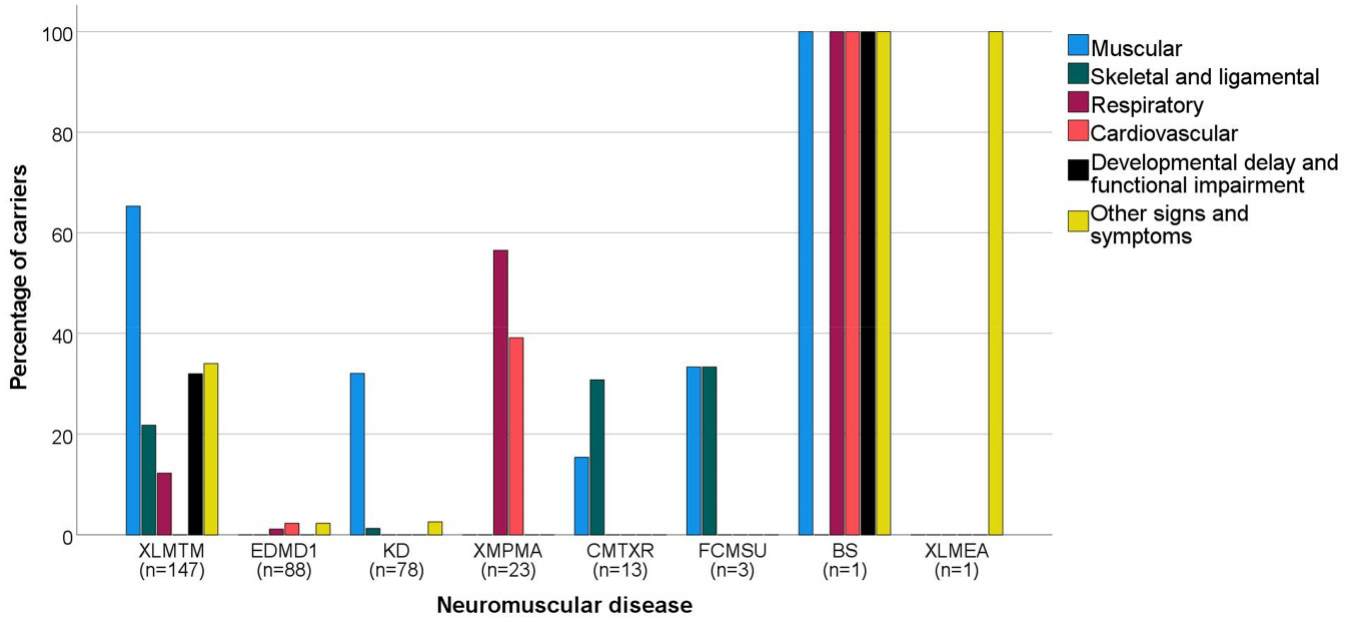
Percentage of carriers with a specific type of sign or symptom per neuromuscular disease (NMD). Signs and symptoms are divided between six groups, with muscular being the farthest left bar in the bar diagram and other signs and symptoms being the farthest right bar in the diagram for each NMD. Below each NMD, the total number of carriers of each disease in this review is displayed (n), with the NMDs being ranked from left to right based on this number. XLMTM: X-linked myotubular myopathy; EDMD1: Emery Dreifuss muscular dystrophy 1; KD: Kennedy's disease; XMPMA: X-linked myopathy with postural muscle atrophy; CMTXR: X-linked recessive Charcot-Marie-Tooth disease; FCMSU: Uruguay faciocardiomusculoskeletal syndrome; BS: Barth syndrome; XLMEA: X-linked myopathy with excessive autophagy.

There were 125 carriers that experienced muscle related signs and symptoms (XLMTM: n = 96 (65%); KD: n = 25 (32%); CMTXR: n = 2 (15%); FCMSU: n = 1 (33%); BS: n = 1 (100%) (Figure 2)).^4,11–36^ These signs and symptoms included observed and reported muscle weakness (including facial weakness such as ptosis), muscle cramps, fasciculations, muscle atrophy and hypertonia. Thirty-eight carriers experienced skeletal and/or ligamental signs and symptoms (XLMTM: n = 32 (22%); KD: n = 1 (1%); CMTXR: n = 4 (31%); FCMSU: n = 1 (33%)).^4,13,18,19,21–24,26,27,30–32,34,36^ Common findings were scoliosis, skeletal asymmetry, joint contractures and carriers experiencing pes cavus. Thirty-three carriers showed respiratory problems (XLMTM: n = 18 (12%); EDMD1: n = 1 (1%); XMPMA: n = 13 (57%); BS: n = 1 (100%)).^4,17,20,27,28,30,35–38^ This consisted of dyspnea, obstructive sleep apnea (OSA), nocturnal hypoventilation and polypnea. Cardiovascular signs and symptoms were described in 12 carriers (EDMD1: n = 2 (1%); XMPMA: n = 9 (39%); BS: n = 1 (100%)) and included hypertension, episodes of chest pain, tachycardia and palpitations.^28,37–39^ 48 carriers manifested developmental delay or functional impairment (XLMTM: n = 47 (32%); BS: n = 1 (100%)).^4,11,13,19,20,22,27–31,36^ Carriers reported a variety of symptoms, including a decreased autonomy, delayed motor milestones and difficulties in daily activities like running, climbing the stairs or rising from the ground. Lastly, we identified other signs and symptoms (XLMTM: n = 50 (34%); EDMD1: n = 2 (2%); KD: n = 2 (3%); BS: n = 1 (100%); XLMEA: n = 1 (100%)).^4,11,13,14,17,19,20,22–24,27,28,31,33,36,37,40,41^ This included lightheadedness, fatigue, tremor, speech problems like dysarthria, urinary incontinence, liver enlargement, pectus excavatum, heart murmur, Babinski's sign and in one case sudden death. In Supplement 3, extensive information about the signs and symptoms per case can be found.

### Findings of ancillary investigations

**Figure 3.**
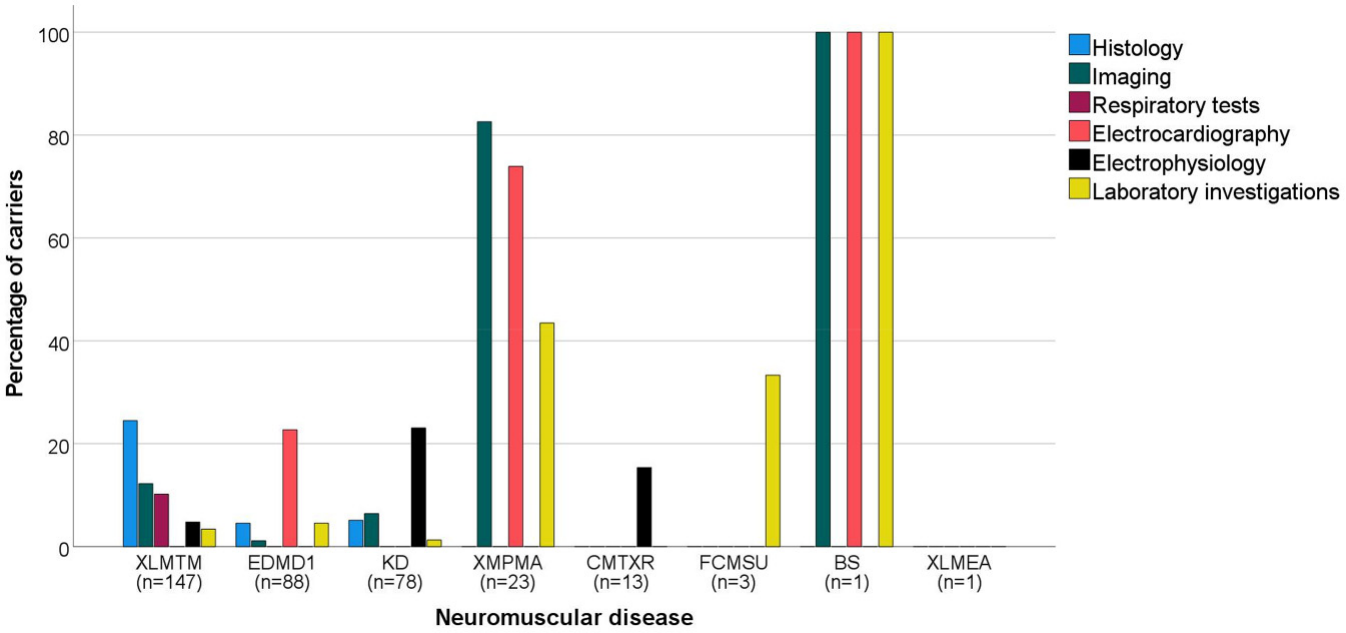
Percentage of carriers with a specific type of abnormal ancillary investigation per neuromuscular disease (NMD). Abnormal ancillary findings are divided between six groups, with histological assessments being the farthest left bar in the bar diagram and laboratory investigations being the farthest right bar in the diagram for each NMD. Below each NMD, the total number of carriers of each disease in this review is displayed (n), with the NMDs being ranked from left to right based on this number. XLMTM: X-linked myotubular myopathy; EDMD1: Emery Dreifuss muscular dystrophy 1; KD: Kennedy's disease; XMPMA: X-linked myopathy with postural muscle atrophy; CMTXR: X-linked recessive Charcot-Marie-Tooth disease; FCMSU: Uruguay faciocardiomusculoskeletal syndrome; BS: Barth syndrome; XLMEA: X-linked myopathy with excessive autophagy.

In total, 44 carriers showed abnormalities in histological assessments (XLMTM: n = 36 (24%); EDMD1: n = 4 (5%); KD: n = 4 (5%) (Figure 3)).^11,13,17,20–27,29,31,33,35,36,42–45^ Muscle biopsies showed altered muscle fibers, often with high fiber size variability, small muscle fibers and abnormal nuclear positioning. Fatty replacement and type 1 fiber predominance were also described. In 44 carriers, abnormalities were found during imaging procedures (XLMTM: n = 18 (12%); EDMD1: n = 1 (1%); KD: n = 5 (6%); XMPMA: n = 19 (83%); BS: n = 1 (100%)).^11,19,20,22,26,28,29,31,36–38,46^ These included computed tomography (CT) and magnetic resonance imaging (MRI) muscle abnormalities like muscle hypertrophy and muscle asymmetry, but also fat infiltration throughout the body. Abnormal echocardiography was reported, mainly in carriers of XMPMA who suffered from hypertrophic cardiomyopathy and reduced diastolic function. Pathological respiratory tests were found in 15 carriers (10%) of XLMTM, which was predominantly described as a reduced vital capacity (VC).^11,19–21,27,36^ Thirty-eight carriers were found to have electrocardiogram (ECG) abnormalities (EDMD1: n = 20 (23%); XMPMA: n = 17 (74%); BS: n = 1 (100%)).^28,37–40,47–50^ ECG findings that were reported most often included first degree atrioventricular (AV) block, sinoatrial (SA) block, atrial fibrillation (AF), QT prolongation, negative T-waves and Q-waves. Twenty-seven carriers were described to have pathological electrophysiology tests (XLMTM: n = 7 (5%); KD: n = 18 (23%); CMTXR: n = 2 (15%)).^15,16,18,20,21,23–26,31–33,45^ High amplitude motor unit potentials, denervation potentials, reduced sensory nerve action potentials and decreased conduction velocity were described. Lastly, abnormal findings in laboratory investigations were reported in 22 carriers (XLMTM: n = 5 (3%); EDMD1: n = 4 (5%); KD: n = 1 (1%); XMPMA: n = 10 (43%); FCMSU: n = 1 (33%); BS: n = 1 (100%)).^15,21,24,27,28,31,34,38,50,51^ This consisted of an elevation of creatine kinase (CK) levels, an elevation of NT-proBNP and one patient with neutropenia. In Supplement 3, extensive information about ancillary investigations per case can be found.

In two articles, neither signs and symptoms nor findings of ancillary investigations were found in the described carriers.^52,53^

## Discussion

This scoping review includes 44 articles on 354 carriers and provides a comprehensive insight into the diverse spectrum of the signs and symptoms that carriers of non-*DMD* XLNMDs exhibit. Through a systematic critical appraisal, all articles were evaluated as being of sufficient quality for inclusion. Muscular signs and symptoms were common among most XLNMDs which may have an effect on everyday life of carriers. Furthermore, ancillary investigations have shown pathological findings in some carriers. Lastly, we found a notable gap in observed cardiovascular symptoms and electrocardiography abnormalities in carriers of EDMD1.

Muscular signs and symptoms were reported in five of the eight XLNMDs included in this review. Signs and symptoms ranged from muscle cramps and atrophy to severe muscle weakness. Interestingly, studies on XLMTM included 47 carriers experiencing developmental delay or functional impairment. Carriers facing challenges with exercise intolerance may experience difficulties in day-to-day life. Muscular symptoms, specifically muscle cramps, are also common in KD. This specific symptom has shown to be a frequent initial symptom in the disease presentation in male patients.^54,55^ Muscle cramps on their own have proven to affect quality of life in other disease.^56^

Skeletal features, such as contractures and scoliosis are relatively common among patients with NMDs and carriers also exhibit these.^57,58^ Pes cavus was seen in several carriers of CMTXR included in this review, which could possibly cause symptoms like pain.^59^ In conjunction with other symptoms or a positive family history, pes cavus could be a reason for clinicians to evaluate a patient more elaborately.^60^

Carriers among four different XLNMDs were noted to have respiratory symptoms. Apart from polypnea and dyspnea, nocturnal hypoventilation and OSA have also been reported. Hypoventilation could be a symptom associated with restrictive respiratory failure.^61^ Non-invasive ventilation could relieve symptoms like daytime tiredness and reduce comorbidity associated with nocturnal hypoventilation and OSA.^62,63^ In addition, respiratory care in *DMD* carriers has proven to improve the threshold of being able to perform daily activities.^64^

Cardiovascular symptoms were seen in EDMD1, XMPMA and BS, all of which are XLNMDs with known cardiac involvement. Symptoms were most severe in a three-year-old BS carrier. EDMD1 carriers reported chest pain or palpitations, while XMPMA carriers experienced hypertension. One EDMD1 carrier likely died from cardiac failure.^37^ Since this female patient did not appear to show symptoms until shortly before her death, this raises concern about the absence of symptoms combined with potentially severe cardiac disease. This finding suggests that sudden death can be the first symptom in EDMD1.^65^

Apart from the study on XLMTM by Reumers et al., pain and fatigue were rarely reported in female carriers with other XLNMDs. Reumers et al. approached carriers directly by conducting a questionnaire study. This may suggest that these symptoms are underreported in clinician-initiated case series without a dedicated patient-survey.^4,66,67^

Patients with XLMTM are known to have structural, myopathic changes in muscle biopsy, including variability in muscle fiber size and abnormal localization of nuclei.^68^ Accordingly, carriers of XLMTM reviewed in this study showed these histopathological findings. Variability in fiber size was also observed in carriers of KD. Larger fiber sizes in patients with XLMTM have been shown to be connected to a better clinical outcome.^69^ Although it poses a substantial challenge to extend this finding to carriers of XLMTM, let alone carriers of other XLNMDs, it may aid in predicting disease progression in the future. However, the invasive nature of performing a biopsy needs to be considered. Muscle imaging may also have prognostic value as shown for other types of NMDs and the high prevalence of muscle affection in *DMD* carrier females.^70,71^ Further imaging in females affected by XLNMDs might help to increase our knowledge on muscle involvement in these conditions.

The gap between reported abnormalities in ECG and the cardiovascular signs and symptoms in carriers of EDMD1 is remarkable. We observed potentially life-threatening heart rhythm abnormalities in some of the studied EDMD1 carriers. In 2017, the American Heart Association (AHA) recommended cardiac follow-up for different NMDs, including EDMD1.^72^ Additionally, carriers of XMPMA not only suffered from various ECG abnormalities, but also showed pathological signs like diastolic dysfunction during echocardiography. These findings could very well be the cause of the observed dyspnea in this carrier group.^38^ These observations regarding cardiac health underscore the importance of identifying and possibly monitoring carriers of XLNMDs.

Abnormalities in electrophysiology were the only sign reported among carriers of CMTXR, although the number of carriers included in this study is low. In terms of laboratory investigations, the elevation of CK levels and NT-proBNP was often mild, which on its own may not be enough to perform further diagnosis on a potential NMD carriership.^73,74^ However, in combination with the signs discussed before, it may have a valuable diagnostic role.

During the 263rd ENMC International Workshop on *DMD* carriers in 2022 it was addressed that labeling symptomatic women as carriers does not adequately represent this cohort. Using the term ‘patients’ would more accurately acknowledge the medical issues and challenges these women face. For this scoping review, we used the term carriers since a consensus on alternative nomenclature for non-*DMD* NMDs has not been reached yet. Nevertheless, the results emphasize that the scientific community should consider addressing these women as patients instead of carriers. This crucial shift may help increase awareness among healthcare professionals and enhance the sense of being taken seriously among affected women. This consideration is even more critical in light of the fact that women who carry *DMD* gene variants tend to neglect their health in order to provide the extensive care needed for their more severely affected child/children.^2^

The strength of this scoping review lies in its wide scope. By systematically including a diverse group of non-*DMD* XLNMDs, this study provides an extensive overview of the signs and symptoms of carriers. Furthermore, this study aligns with recent developments in the neuromuscular field: for instance, females with XLMTM were included in the recent Dynacure trial. Moreover, the Duchenne Awareness Day in 2022 was focused on females.

This review has several limitations. Non-*DMD* XLNMD carriers are understudied and brain involvement – though relevant to quality of life – is not included. The rarity of these NMDs limits the carrier pool, restricting broader conclusions, particularly for XLMEA, BS and FCMSU.

Next, distinguishing between signs and symptoms in this study was challenging, particularly for muscle weakness. It can be a symptom when self-reported or a sign when objectively measured using the MRC scale. As many articles lacked objective measurements, we combined reported and observed muscle weakness in one graph. Acknowledging this ambiguity, we aimed to address it thoughtfully in this review.

Publication bias is likely, as studies may not report asymptomatic carriers, making it difficult to estimate symptom prevalence in the general carrier population. Selection bias could also arise during screening and data extraction due to variations in interpreting inclusion and exclusion criteria. To minimize this, all screening decisions were made by at least two independent reviewers.

## Conclusion

In short, carriers of XLNMDs, including *DMD*, can exhibit signs and symptoms, impacting their quality of life. This review highlights the broad clinical spectrum of non-*DMD* XLNMD carriers and recommends referring to them as patients rather than carriers. It provides clinicians with insights into their presentation, aiding diagnosis and treatment. Further research is needed to better estimate prevalence and daily life impact.

## Supplemental Material

sj-sav-1-jnd-10.1177_22143602251330441 - Supplemental material for Signs and symptoms of carriers of non-*DMD* X-linked neuromuscular diseases: A scoping review Supplemental material, sj-sav-1-jnd-10.1177_22143602251330441 for Signs and symptoms of carriers of non-*DMD* X-linked neuromuscular diseases: A scoping review by Job Simons, Amanda Dekker, Rosanne Govaarts, Anna Sarkozy, Christian Windpassinger, Saskia Houwen and Nicol Voermans in Journal of Neuromuscular Diseases

sj-docx-2-jnd-10.1177_22143602251330441 - Supplemental material for Signs and symptoms of carriers of non-*DMD* X-linked neuromuscular diseases: A scoping review Supplemental material, sj-docx-2-jnd-10.1177_22143602251330441 for Signs and symptoms of carriers of non-*DMD* X-linked neuromuscular diseases: A scoping review by Job Simons, Amanda Dekker, Rosanne Govaarts, Anna Sarkozy, Christian Windpassinger, Saskia Houwen and Nicol Voermans in Journal of Neuromuscular Diseases

sj-xlsx-3-jnd-10.1177_22143602251330441 - Supplemental material for Signs and symptoms of carriers of non-*DMD* X-linked neuromuscular diseases: A scoping review Supplemental material, sj-xlsx-3-jnd-10.1177_22143602251330441 for Signs and symptoms of carriers of non-*DMD* X-linked neuromuscular diseases: A scoping review by Job Simons, Amanda Dekker, Rosanne Govaarts, Anna Sarkozy, Christian Windpassinger, Saskia Houwen and Nicol Voermans in Journal of Neuromuscular Diseases

sj-xlsx-4-jnd-10.1177_22143602251330441 - Supplemental material for Signs and symptoms of carriers of non-*DMD* X-linked neuromuscular diseases: A scoping review Supplemental material, sj-xlsx-4-jnd-10.1177_22143602251330441 for Signs and symptoms of carriers of non-*DMD* X-linked neuromuscular diseases: A scoping review by Job Simons, Amanda Dekker, Rosanne Govaarts, Anna Sarkozy, Christian Windpassinger, Saskia Houwen and Nicol Voermans in Journal of Neuromuscular Diseases
