# Supplementary material for: Signs and symptoms of carriers of non-DMD X-linked neuromuscular diseases: A scoping review
Source: J Neuromuscul Dis. 2025 Mar 29;12(4):473–86. doi: 10.1177/22143602251330441 (PMC13142866; doi:10.1177/22143602251330441)
Supplement: sj-docx-2-jnd-10.1177_22143602251330441 - Supplemental material for Signs and symptoms of carriers of non-DMD X-linked neuromuscular diseases: A scoping review [file sj-docx-2-jnd-10.1177_22143602251330441.docx]

**PubMed (3023 hits, 22 September 2023)**

((("heterozygote"[MeSH Terms] OR "genetic diseases, x linked"[MeSH:noexp] OR "Genes, X-Linked"[Mesh] OR "x-linked"[Title/Abstract])

AND

("Motor Neuron Disease"[Mesh:noexp] OR "Muscular Diseases"[Mesh] OR "Neuromuscular Junction Diseases"[Mesh] OR "Hereditary Sensory and Motor Neuropathy"[Mesh] OR "neuromuscular"[Title/Abstract] OR "myopath*"[Title/Abstract]))

OR

("bulbo spinal atrophy, x linked"[MeSH] OR "X-Linked Emery-Dreifuss Muscular Dystrophy"[Mesh] OR "Myopathies, Structural, Congenital"[Mesh] OR Emery-Dreifuss Muscular Dystroph*[tiab] OR Kennedy Disease*[tiab] OR Kennedy's Disease*[tiab] OR Kennedy syndrome*[tiab] OR Bulbo-Spinal Atroph*[tiab] OR bulbospinal atroph*[tiab] OR spinobulbar muscular atroph*[tiab] OR Charcot-Marie-Tooth Disease*[tiab] OR "Neuroacanthocytosis, Mcleod Type" [Supplementary Concept] OR "Spinal Muscular Atrophy, Distal, X-Linked 3" [Supplementary Concept] OR Hereditary X-Linked Recessive Spastic Paraplegia [tiab] OR X Linked Recessive Hereditary Spastic Paraplegia [tiab] OR Mcleod syndrome*[tiab] OR Barth Syndrome*[tiab] OR X-linked Myotubular Myopathy [tiab] OR X linked myotubular myopathy [tiab] OR x-linked centronuclear myopath*[tiab] OR X-linked myopathy with excessive autophagy [tiab] OR X linked myopathy with excessive autophagy [tiab] OR X-linked Myopathy with Postural Muscle Atrophy [tiab] OR X linked Myopathy with Postural Muscle Atrophy [tiab]))

AND

("Signs and Symptoms"[Mesh] OR symptom[tiab] OR symptoms[tiab] OR characteri*[tiab] OR sign[tiab] OR signs[tiab] OR clinical[tiab])

AND

(Female[mesh] OR female*[tiab] OR carrier[tiab] OR carriers[tiab])

**Embase (3687 hits, exclude limit to conference abstract, 22 September 2023)**

(((heterozygote/ or x chromosome recessive disorder/ or "x-linked".ti,ab,kf.)

AND

(Motor Neuron Disease/ or muscle disease/ or muscle atrophy/ or muscle contracture/ or muscle fatigue/ or muscle hypertrophy/ or muscle malformation/ or exp muscle rigidity/ or muscle strain/ or muscle tightness/ or myalgia/ or myofibrosis/ or myositis/ or neuromuscular disease/ or rhabdomyolysis/ or fibromuscular dysplasia/ or delayed onset muscle soreness/ or myofascial pain/ or neuromuscular disease/ or anterior horn cell disease/ or hereditary motor sensory neuropathy/ or exp myopathy/ or exp neuromuscular junction disorder/ or muscular dystrophy/ or distal myopathy/ or "neuromuscular".ti,ab,kf. or "myopath*".ti,ab,kf.))

OR

(emery dreifuss muscular dystrophy/ or kennedy disease/ or centronuclear myopathy/ or Emery-Dreifuss Muscular Dystroph*.ti,ab,kf. or Kennedy Disease*.ti,ab,kf. or Kennedy's Disease*.ti,ab,kf. or Bulbo-Spinal Atroph*.ti,ab,kf. or Barth Syndrome*.ti,ab,kf. or Hereditary Spastic Paraplegia.ti,ab,kf. or Kennedy syndrome*.ti,ab,kf. or bulbospinal atroph*.ti,ab,kf. or spinobulbar muscular atrophy*.ti,ab,kf. or Charcot-Marie-Tooth Disease*.ti,ab,kf. or X-linked Myopathy with Excessive Autophagy.ti,ab,kf. or Mcleod syndrome*.ti,ab,kf. or distal Spinal Muscular Atroph*.ti,ab,kf. or x-linked Spinal Muscular Atroph*.ti,ab,kf. or Hereditary X-Linked Recessive Spastic Paraplegia.ti,ab,kf. or X-Linked Recessive Hereditary Spastic Paraplegia.ti,ab,kf. or X-linked Myotubular Myopathy.ti,ab,kf. or x-linked centronuclear myopath*.ti,ab,kf. or X-linked myopathy with postural muscle atrophy.ti,ab,kf.))

AND

(symptomatology/ or clinical feature/ or symptom/ or symptom.ti,ab,kf. or symptoms.ti,ab,kf. or characteri*.ti,ab,kf. or sign.ti,ab,kf. or signs.ti,ab,kf. or clinical.ti,ab,kf.)

AND

(female/ or girl/ or female*.ti,ab,kf. or carrier.ti,ab,kf. or carriers.ti,ab,kf.)

**Web of Science (501 hits, 22 September 2023)**

TS= (

(((heterozygote OR x-linked)

AND

(“Motor Neuron Disease*” OR "Muscular Disease*" OR "Hereditary Sensory and Motor Neuropath*" OR "neuromuscular" OR "myopath*" OR "bulbo spinal atrophy*” OR “Emery-Dreifuss Muscular Dystroph*” OR “bulbospinal atroph*” OR “spinobulbar muscular atrophy*” OR "Spinal Muscular Atroph*”))

OR

(“Kennedy Disease*” OR “Kennedy's Disease*” OR “Kennedy syndrome*” OR Charcot-Marie-Tooth Disease* OR “Mcleod syndrome*” OR “Hereditary X-Linked Recessive Spastic Paraplegia” OR “X Linked Recessive Hereditary Spastic Paraplegia” OR “Barth Syndrome*” OR “X-linked Myotubular Myopathy” OR “x-linked centronuclear myopath*” OR “X-linked Myopathy with Excessive Autophagy” OR “X-linked Myopathy with Postural Muscle Atrophy”))

AND

(symptom OR symptoms OR characteri* OR sign OR signs OR clinical)

AND

(female* OR carrier OR carriers)

)
